# Phloem Regeneration Is a Mechanism for Huanglongbing-Tolerance of “Bearss” Lemon and “LB8-9” Sugar Belle^®^ Mandarin

**DOI:** 10.3389/fpls.2019.00277

**Published:** 2019-03-20

**Authors:** Honghong Deng, Diann Achor, Ed Exteberria, Qibin Yu, Dongliang Du, Daniel Stanton, Guolu Liang, Fred G. Gmitter Jr.

**Affiliations:** ^1^ College of Horticulture and Landscape Architecture, Southwest University, Chongqing, China; ^2^ Citrus Research and Education Center, University of Florida, Lake Alfred, FL, United States

**Keywords:** HLB, citrus greening, anatomy, phloem disruption, replacement phloem, disease tolerance

## Abstract

Huanglongbing (HLB) is an extremely destructive and lethal disease of citrus worldwide, presumably caused by phloem-limited bacteria, *Candidatus* Liberibacter asiaticus (*C*Las). The widespread invasiveness of the HLB pathogen and lack of natural HLB-resistant citrus cultivars have underscored the need for identifying tolerant citrus genotypes to support the current citrus industry’s survival and potentially to lead to future natural HLB resistance. In this study, transverse sections of leaf lamina and midribs were examined with light and epifluorescence microscopy to determine anatomical characteristics that underlie HLB-tolerant mechanisms operating among “Bearss” lemon, “LB8-9” Sugar Belle^®^ mandarin, and its sibling trees compared with HLB-sensitive “Valencia” sweet orange. The common anatomical aberrations observed in all *C*Las-infected varieties are as follows: phloem necrosis, hypertrophic phloem parenchyma cells, phloem plugging with abundant callose depositions, phloem collapse with cell wall distortion and thickening, excessive starch accumulation, and sometimes even cambium degeneration. Anatomical distribution of starch accumulation even extended to tracheid elements. Although there were physical, morphological, and pathological similarities in the examined foliage, internal structural preservation in “Bearss” lemon and “LB8-9” Sugar Belle^®^ mandarin was superior compared with HLB-sensitive “Valencia” sweet orange and siblings of “LB8-9” Sugar Belle^®^ mandarin. Intriguingly, there was substantial phloem regeneration in the tolerant types that may compensate for the dysfunctional phloem, in comparison with the sensitive selections. The lower levels of phloem disruption, together with greater phloem regeneration, are two key elements that contribute to HLB tolerance in diverse citrus cultivars.

## Introduction

Citrus Huanglongbing (HLB, previously called citrus greening and yellow dragon disease), an extremely destructive and lethal disease of citrus (Bové, 2006), was discovered in Guangdong province in south China in 1919 (Reinking, 1919). Since first detected in Miami-Dade county of south Florida in 2005 (Halbert, 2005), fruit yields have declined annually, resulting in substantial economic losses according to the USDA’s National Agricultural Statistics Service (NASS) (Ferreira and Perez, 2017). The sharp decline in fruit production is a consequence of the widespread invasiveness of the pathogen, the unavailability of curative treatments, and the lack of HLB-resistant cultivars (Bové, 2006; da Graca et al., 2016; Miles et al., 2017).

The putative pathological agent of HLB, *Candidatus* Liberibacter spp., is a Gram-negative, thin-walled, phloem-limited bacterial genus belonging to α (alpha) subdivision of the class *Proteobacteria* (Jagoueix et al., 1994; Bové, 2006). So far it has not been successfully cultured. Currently, three species are recognized worldwide: *Ca*. *L. asiaticus* (*C*Las) (Bové, 2006), *Ca*. *L. africanus* (*C*Laf) (Jagoueix et al., 1994), and *Ca*. *L. americanus* (*C*Lam) (Teixeira et al., 2005), on the basis of their eco-geographic range, transmission vector, and adaptation to warmer or cooler environments. In Florida, *C*Las is the only pathogen identified (Wang and Trivedi, 2013; Brodersen et al., 2014), with primary transmission by the Asian citrus psyllid (ACP, *Diaphorina citri*) (Bové, 2006), though secondary transmission is possible by grafting and dodder (*Cuscuta pentagona*) (Zhou et al., 2007; da Graça, 2008). Foliar asymmetrical chlorosis and blotchy mottle appearance is the most recognized characteristic of HLB symptomatology, which also includes yellow foliage and shoots, leaf loss and fruit drop, stunting and twig dieback, premature and lopsided fruits, and eventual tree death in some, but not all, situations (Aritua et al., 2013; da Graca et al., 2016). Recently, it has been reported that HLB has swept through almost 100% of commercial citrus groves in Florida (Browning, 2015), and more than 80% of all citrus trees have been affected (Albrigo and Stover, 2015).

Previous evidence indicates that *C*Las always resides and colonizes the sieve tubes within citrus phloem tissue (Jagoueix et al., 1994; Kim et al., 2009; Aritua et al., 2013; Wang et al., 2017), which is responsible for carrying photosynthates from source-to-sink in plants (Heo et al., 2014). It is this very tissue that contains the essential and massive nutrient-rich components that support the life activities of both *C*Las and ACP (Hijaz et al., 2016). At the anatomical level, citrus leaf tissue exhibited conspicuous changes induced by *C*Las infection compared to healthy foliage. Ultrastructural examination of tissue from *C*Las-inoculated sweet orange [*Citrus sinensis* (L.) Osbeck] and grapefruit (*C. paradisi* MacFadyen) revealed the early histological symptomatology of middle lamella swelling between cell walls around sieve elements (Folimonova and Achor, 2010), also described as phloem necrosis (Schneider, 1967; Achor et al., 2010). Phloem necrosis is usually accompanied by phloem sieve element plugging from abundant callose and phloem protein 2 deposition (Achor et al., 2010; Koh et al., 2012; Albrigo et al., 2014), followed by phloem cell wall distortion and sieve element collapse (Etxeberria and Narciso, 2015). Because of these phloem disruptions, transport of photosynthates is severely obstructed (Aritua et al., 2013; Albrigo et al., 2014; Etxeberria and Narciso, 2015), which in turn may be responsible for the accumulation of abnormally large quantities of starch granules in virtually all living cells of the aerial organs (Schneider, 1968; Etxeberria et al., 2009), including phloem parenchyma cells and sieve elements (Folimonova and Achor, 2010; Gonzalez et al., 2012).

Currently, there are no commercial citrus cultivars, varieties or scion-rootstock grafting combinations with natural resistance to *C*Las infection (da Graca et al., 2016; Wang et al., 2017). However, a few commercial varieties such as lemon [*C. limon* (L.) Burm. F.] and Persian lime (*C. latifolia*), along with US-897 rootstock (*C. reticulata* Blanco × *Poncirus trifoliata* L. Raf.) and the “LB8-9” Sugar Belle^®^ mandarin hybrid (SB; “Clementine” mandarin × “Minneola” tangelo) (Gmitter et al., 2010) have shown apparent HLB tolerance under Florida natural HLB-endemic conditions (Albrecht and Bowman, 2011; Ramadugu et al., 2016; Stover et al., 2016; Killiny et al., 2017; Miles et al., 2017; Wang et al., 2017). SB mandarin and “Bearss” lemon trees in various locations in Florida maintain vigorous growth, and fruit yield is not significantly affected by *C*Las infection (Gmitter et al., unpublished data). “Valencia” sweet orange (*C. sinensis* (L.) Osbeck) is a well-known HLB-sensitive cultivar (Folimonova et al., 2009). LB8-1, LB8-2, LB8-15, and LB9-13 mandarins are the siblings of SB mandarin; however, they have been found to be very sensitive to HLB, based on more than 10 years of observation (Gmitter et al., unpublished data). Orange, mandarin, and lemon represent three different kinds of citrus from the taxonomic point of view (Wu et al., 2018). Taken together, these similarities and differences in the botanical origin and the range of sensitivity to HLB provide an excellent opportunity to identify factors impacting HLB tolerance.

With overall citrus production sharply down in the United States and other major citrus-producing countries worldwide (Ferreira and Perez, 2017), identifying the presence of HLB tolerance in citrus germplasm resources is highly needed, crucial to the current citrus industry’s survival, and is indispensable for future ultimate natural HLB resistance (Killiny et al., 2017; Miles et al., 2017). In the present work, we conducted a comparative pathological and anatomical investigation by sectioning the lamina and midribs and examination by light and epifluorescence microscopy to understand what interior structure makes “Bearss” lemon and SB mandarin tolerant and others sensitive to *C*Las infection in the natural field environment. Results from this study may provide a very useful supplement to current HLB tolerant citrus germplasm knowledge. We hope that in future work, these observations and HLB tolerance mechanism can be used to modify and create citrus germplasm that is more tolerant or ultimately resistant to HLB.

## Materials and Methods

### Plant Materials

**Figure 1:**
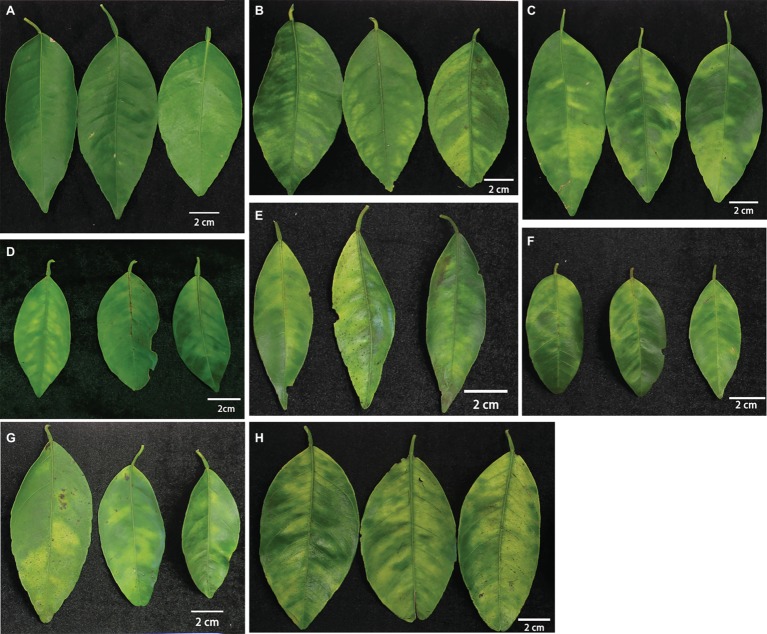
The materials used in this study. **(A)** HLB negative and greenhouse-grown SB mandarin; **(B–H)** blotchy mottle leaves from **(B)** “Bearss” lemon; **(C)** SB mandarin; **(D)** “Valencia” sweet orange; **(E)** LB8-1 mandarin; **(F)** LB8-2 mandarin; **(G)** LB8-15 mandarin; and **(H)** LB9-13 mandarin. Notes: LB8-1, LB8-2, LB8-15, and LB9-13 mandarin are siblings of SB, having common parents, “Clementine” mandarin (*C. reticulata* Hort. ex Tanaka) × “Minneola” tangelo [“Duncan” grapefruit (*C. paradisi* Mac.)] × [“Dancy” tangerine (*C. reticulata*)]. The leaves of each cultivar were captured by digital camera with a scale bar of 2 cm.

For basic anatomical studies, fully expanded and hardened leaves of spring flushes with typically visual blotchy mottle symptoms were sampled at the end of July from “Bearss” lemon, SB mandarin, “Valencia” sweet orange, and siblings of SB mandarin (LB8-1, LB8-2, LB8-15, and LB9-13). SB mandarin and siblings and “Valencia” sweet orange trees were grown in an experimental field of UF-CREC, and “Bearss” lemon trees were grown in a commercial orchard near Vero Beach, Florida. All trees were naturally exposed to HLB disease for at least 10 years. The experimental trees were approximately 20 years old. Correspondingly, HLB-free control SB mandarin samples were collected from a secured screen-house at CREC, which was thoroughly protected from infection and exposed to the same natural environment with those HLB-affected trees. The foliar samples were randomly selected from mature spring flushes with similar physical age and HLB status (Figure 1). Before sampling, the trees were assessed by real-time qPCR (quantitative polymerase chain reaction) to confirm *C*Las infection, according to Li et al. (2006).

### Sample Preparation

The leaves were collected between 9:00 and 10:00 am on a sunny day. Leaf midrib tissue was dissected into 2–3 mm segments with single edged disposable blades and placed immediately into fixative solution of 4% paraformaldehyde and 1% glutaraldehyde in 0.1 M Sorensen’s phosphate buffer (pH 7.2) to preserve structure. The tissue was fixed at a minimum of 4 h at RT (room temperature), or overnight at 4°C to adequately infiltrate the specimens. Once fixed, the samples were thoroughly rinsed using the above buffer. Paraffin wax is water immiscible. Therefore, the specimens were dehydrated with ethanol solutions of an increasing concentration (30, 50, 70, 85, 95–100% ethanol) to remove both bound and free water. Ethanol changes were made every hour. The successive incremental concentration was used to avoid exorbitant distortion of the specimens.

To further dehydrate the tissue, three changes of increasing ratio of TBA (tertiary buty1 alcohol, also called tert-butanol) were used to displace the ethanol in the tissue (3:1, 1:1, 1:3—ethanol: TBA). Tissue was rinsed in 100% TBA for an hour. TBA was gradually replaced with previously melted paraffin wax using a similar ratio (3:1, 1:1, 1:3—TBA: Paraffin) in an oven at 58°C. Solution changes occurred every 8–14 h. Once transitioned to 100% paraffin, an additional three paraffin changes were made every 24 h for optimal infiltration. Tissue was embedded in 100% paraffin using embedding base mold in the desired orientation. Paraffin was allowed to harden at room temperature and then molds were incubated at 4°C overnight. Paraffin blocks were stored at room temperature. The blocks were then sectioned using a Leica 2155 microtome. 10 μm sections were cut to form a ribbon. Sections were placed on a drop of water on a microscope slide and placed on a hot plate at 37°C hotplate overnight to ensure firm adhesion of sections to the slides.

### Methylene Blue-Azure II-Basic Fuchsin Staining

Slides were dewaxed using three changes of Histoclear II for 15 min each, followed by rehydration through a series of decreasing ethanol washes (100, 70, 30%, 5 min per wash). Slides were rinsed in ultrapure water before staining by methylene blue-azure II-basic fuchsin (Humphrey and Pittman, 1974). The procedures were optimized to stain with methylene blue/azure A for 45 s and counterstaining with basic fuchsin for 30 s, followed by a thorough rinse with distilled water. Slides were dehydrated in an ethanol series (30, 70, 100%) for 3 min each and then cleared in three changes of Histoclear II. A drop of Cytoseal mount was placed over the sections, and a coverslip was placed on the slide. Images were captured using an OMAX 14.0MP CCD digital camera attached to an Olympus BX61 epifluorescence microscope (Olympus Inc., Tokyo, Japan).

### Data Analysis

The scale bars were also captured under the same magnification with samples as reference for the following phloem strip width standardization. The phloem and replacement phloem strip width were quantitatively measured using Image J software (National Institutes of Health, Wayne Rasband, Maryland, USA). The ratio of replacement phloem is calculated by dividing the width of replacement phloem by the total phloem width of the maximal portion within the same leaf. The same position of all the leaves was measured. The result of each leaf was the average of three times technical replicates. The ratio of replacement phloem of each cultivar was based on the average of at least 35 leaves. Minitab version 17 software was used for the means and standard errors calculation. Differences within cultivars were statistically evaluated by one-way ANOVA analysis (analysis of variance). Subsequent comparisons were made using Tukey’s HSD test with 95% confidence interval.

## Results

### Comparative Anatomical Changes of HLB-Affected Citrus Lamina

**Figure 2:**
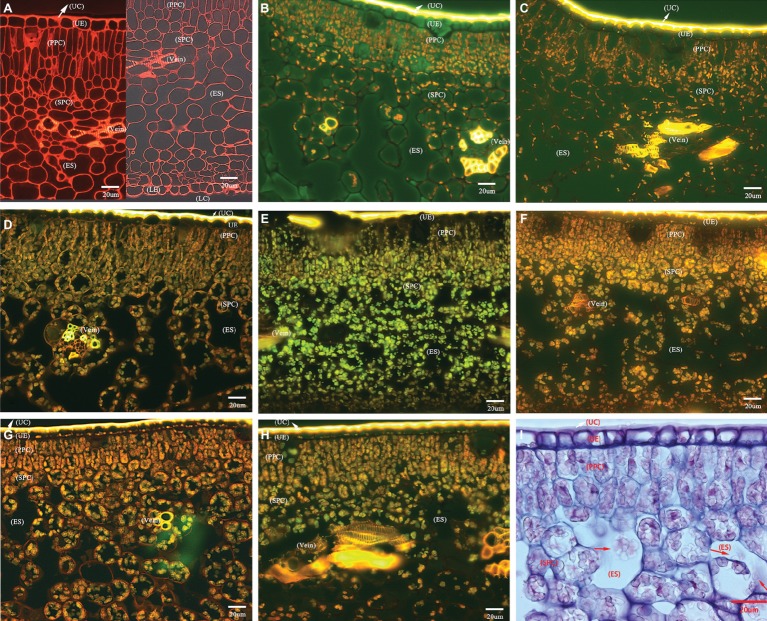
Photomicrographs of transverse section showing comparative anatomical changes of HLB-affected citrus lamina. **(A–H)** Epifluorescence micrograph of anatomical characters through 20× objective lens. **(A)** HLB-free SB mandarin; **(B)** “Bearss” lemon; **(C)** SB mandarin; **(D)** “Valencia” sweet orange; **(E)** LB8-1 mandarin; **(F)** LB8-2 mandarin; **(G)** LB8-15 mandarin; **(H)** LB9-13 mandarin; and **(I)** light micrograph through 40× objective lens with methylene blue-azure A solution and basic fuchsin solution staining, LB8-15 mandarin. The red arrow in image **(I)** indicates the starch granules in a light micrograph. Scale bars = 20 μm. Abbreviations: UC, upper cuticle; UE, upper epidermis; PPC, palisade parenchyma cell; SPC, spongy parenchyma cell; ES, intercellular air empty space; LE, lower epidermis; and LC, lower cuticle.

The transverse sections of the lamina internal structures are presented in Figure 2. Based on many repeated microscopic observations, there are basic anatomical similarities in the lamina common in HLB-affected and HLB-free citrus leaves (Figure 2). The internal structure is protected by an upper and lower epidermis, which typically consists of one thick and compressed uniseriate cell layer. Anatomically, the epidermis is covered by a thick layer of slightly undulate cuticle on the adaxial and abaxial surface. The stomata are found on the abaxial surface. Directly below the epidermis, there stand in parallel columns two or three elongated and pillar-shaped layers of photosynthetic palisade parenchyma cells. Beneath the palisade parenchyma cells lies the loosely packed and irregularly shaped spongy parenchyma cells. Some visible veins, composed of vascular tissue with the xylem above the phloem, are found immersed in lamina parenchyma cells (Figure 2A).

HLB-free control SB mandarin possessed very little or nonexistent starch accumulation in lamina internal structure (Figure 2A). Among the HLB-affected citrus varieties, however, the lamina of all cultivars displayed starch accumulation but with differing severity. The starch grains were scattered rather sporadically and rarely in the epidermal cells of “Bearss” lemon and SB mandarin lamina (Figures 2B,C); however, they were observed as a continuous layer settled to the bottom of the adaxial epidermis in HLB-sensitive “Valencia” sweet orange and SB mandarin siblings (Figures 2D–H, note the conspicuous starch grain layer in LB8-2, LB8-15, and LB9-13 mandarins in Figures 2F–H). A number of large, spherical or oblate spheroid starch granules entirely filled the whole volume of most palisade parenchyma cells, and the spongy parenchyma cell matrix in “Valencia” sweet orange (Figure 2D) and SB mandarin siblings (Figures 2E–I). Few and partially-filled starch granules, by contrast, were observed in “Bearss” lemon and SB mandarin mesophyll cells (Figures 2B,C).

### Pathological Anatomy Modifications of Citrus Midribs Induced by HLB

**Figure 3:**
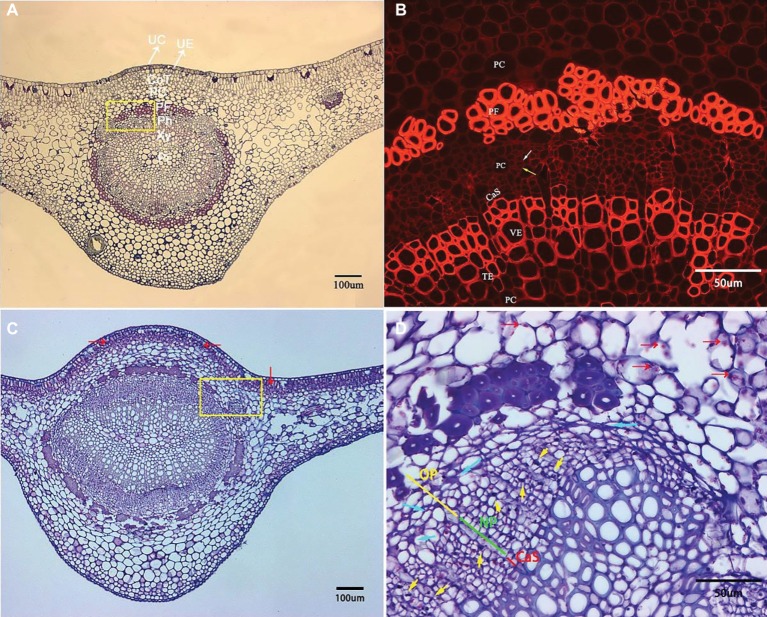
Transverse section micrograph of leaf midrib tissue from healthy and HLB-affected SB mandarin tree. Midrib section was observed, and photographs taken under epifluorescence **(B)** and light microscopy **(A,C,D)**. **(B)** and **(D)** are the magnification of the yellow rectangular frame in **(A)** and **(C)**, respectively. **(A,B)** HLB-free SB mandarin. The white arrow indicates the sieve tube elements, and the yellow arrow indicates the companion cell. Healthy phloem cells have smooth edges, thinner cell walls, and lack starch grains. **(C,D)** HLB-affected SB mandarin showing phloem plugging with abundant callose depositions (yellow arrows), phloem collapse with cell wall distortion and thickening (blue arrows), and starch accumulation (red arrows). The green line indicates the replacement phloem, the yellow line indicates peripheral old phloem, and the red line indicates cambium strip. Abbreviations: UC, upper cuticle; UE, upper epidermis; CoT, collenchyma tissue; PC, parenchyma cell; PF, phloem fibre; Ph, phloem; Xy, xylem; Pi, pith; CaS, cambium strip; VE, vessel elements; TE, tracheid elements; NP, new phloem also called replacement phloem in this manuscirpt; and OP, peripheral old phloem. Scale bars = 100 μm **(A,C)**; scale bars =50 μm **(B,D)**.

Microscopic examination of the transverse sections of the prominent midribs of citrus leaves is presented in Figure 3. From the adaxial to abaxial side of the midrib, collenchyma tissue occasionally was found below the upper epidermis. The largest vascular bundle is embedded in the central space with xylem being positioned toward the inner boundary and phloem toward outer boundary, which run parallel to each other and are surrounded by an incomplete sclerenchyma ring (phloem fibers) with heavily thickened cell walls. Xylem tissue is composed of vessel elements (large), tracheid elements (small), and parenchyma cells (Figure 3A). Within phloem tissue, the parenchyma cells can be easily recognized with the largest cell size. Each phloem sieve element (SE) is associated with a very small and neighboring companion cell (CC) that supports the functioning of SEs (Figure 3B, white and yellow arrows, respectively).

Compared to the basic structure of foliar phloem tissue from healthy trees, the anatomical aberrations observed in the foliar phloem cells of HLB-affected SB trees are as follows: a few phloem cells plugged with abundant callose depositions (Figure 3D, yellow arrows), phloem collapse with cell wall distortion and thickening (Figure 3D, blue arrows), and starch accumulation (Figures 3C,D, red arrows). Before metaphloem elements are entirely collapsed, SB mandarin showed obvious phloem regeneration (Figure 3D, the phloem regeneration was indicated by green line).

### Differential Anatomical Changes in Foliar Phloem of HLB-Affected Citrus Cultivars

**Figure 4:**
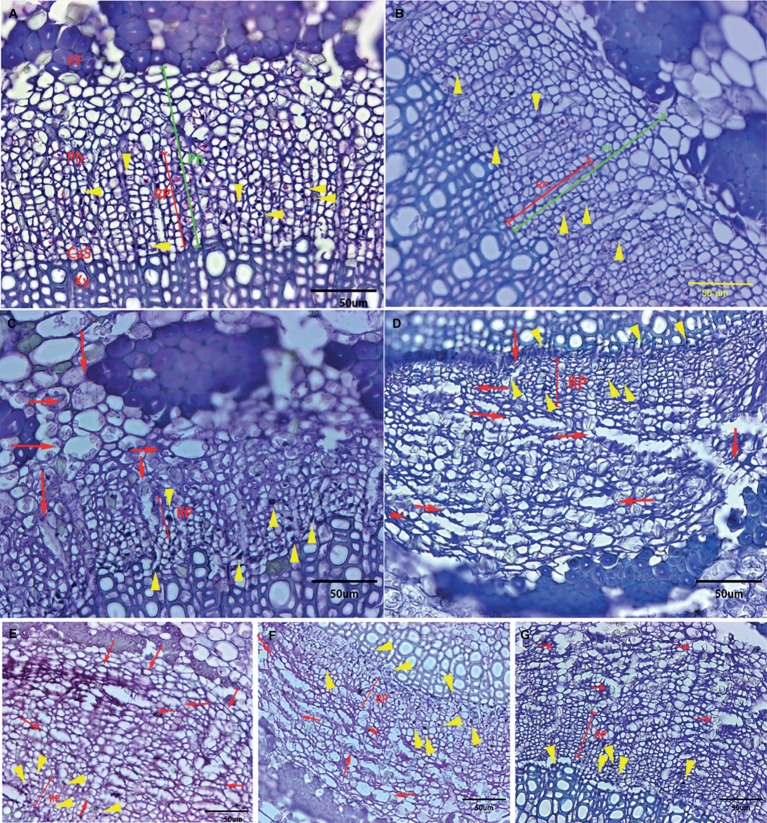
Transverse section showing anatomical changes of leaf midrib in response to *C*Las infection among different citrus cultivars. Midrib section was observed and photographs taken under light microscope. **(A)** “Bearss” lemon; **(B)** SB mandarin; **(C)** “Valencia” sweet orange; **(D)** LB8-1 mandarin; **(E)** LB8-2 mandarin; **(F)** LB8-15 mandarin; and **(G)** LB9-13 mandarin. The red arrow indicates the hypertrophic parenchyma cells that became abnormally enlarged or misshapen to giant cells. The callose-plugged sieve elements appeared as densely stained blue or black spots as indicated by yellow arrows. The red line segment indicates the replacement phloem. The green segment indicates the phloem. Abbreviations: PF, phloem fibre; Ph, phloem; CaS, cambium strip; RP, replacement phloem; and Xy, xylem. Scale bar = 50 μm.

Although all the examined midribs came from spring flush leaves of similar age and *C*Las infection, confirmed by qPCR, the severity of anatomical aberrations induced by *C*Las infection varied. On the basis of comparison to the normal parenchyma cells that have smooth edges, “Valencia” sweet orange and SB mandarin siblings suffered more severe cell necrosis, which was characterized by excessively swollen or hypertrophic phloem parenchyma cells. The hypertrophic parenchyma cells of “Valencia” sweet orange and SB mandarin siblings became abnormally enlarged or misshapen to giant cells (see the red arrows in Figures 4C–G), while the phloem parenchyma cells of “Bearss” lemon and SB mandarin remained largely unchanged (Figures 4A,B).

**Figure 5:**
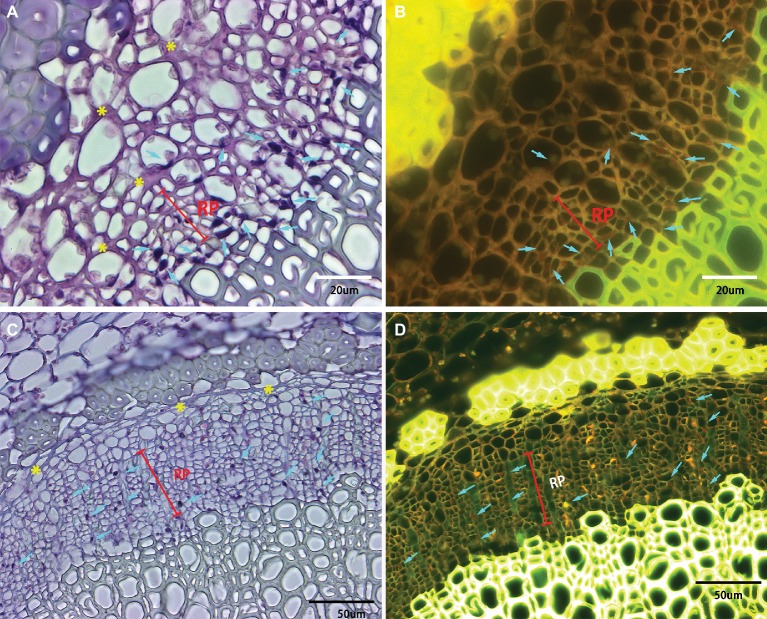
Light and epifluorescence photomicrographs of phloem plugging and collapse. Phloem plugging by callose deposition was marked by the light blue arrows with the dark blue or black densely staining spots lying toward the inner side of sieve tubes. The darkly stained strip resembling a solid barrier oriented tangentially, radially, or irregularly between adjacent phloem cells represented phloem collapse (indicated by yellow asterisks). **(A,B)** Valencia sweet orange, through 40× objective lens, **(C,D)** Sugar Belle mandarin, through 20× objective lens. Note a high accumulation of starch in the parenchyma cells and phloem cells. Scale bars = 20 and 50 μm.

Phloem plugging is one of the anatomical modifications and disorders in sieve elements of all the examined HLB-affected cultivars (Figures 3D, 4, 5). Amorphous plugging was not observed in HLB-free control tissues (Figure 3A). The dark blue or black densely staining spots lying toward the inner side of sieve tubes seen in the light micrographs, resulting from methylene blue-azure II-basic fuchsin staining, represent the phloem plugging with callose deposition (Figures 3D, 4, yellow arrows; Figures 5A,C, blue arrows). There were significant amounts of callose deposition located in the sieve elements of new or replacement phloem, or adjacent to cambium regions, in all the cultivars (Figures 4, 5A,C). Callose depositions were identically confirmed by the epifluorescence micrograph observations by the appearance of red fluorescence spreading prolifically in sieve elements of replacement phloem or adjacent to cambium regions (Figures 5B,D). Some of the sieve elements were entirely covered with an abundance of amorphous callose deposition (Figures 5A,B).

Phloem collapse with cell wall distortion and thickening is much more frequently observed in “Valencia” sweet orange (Figures 4C, 5A) and SB siblings (Figures 4D–G). Peripheral old sieve tube members and companion cells collapsed into darkly stained strips and chromophilic masses resembling a solid barrier oriented tangentially, radially, or irregularly between adjacent phloem cells (Figure 5). Even at higher magnifications of 20× objective lens, the individual cells are not easily distinguished because of the appearance of dark purple or blue strips of partial or total phloem collapse and disintegration (Figures 4C–G). Among the collapsed phloem cells, sieve elements and companion cells were significantly collapsed, while phloem parenchyma cells were expanded to a larger size (Figure 5). The amount of collapsed phloem in “Valencia” sweet orange and SB siblings was much greater than that in “Bearss” lemon or SB mandarin (Figures 4, 5). By contrast, no phloem collapse was seen in the midribs of HLB-free SB mandarin (Figure 3A).

A particularly intriguing result is that, as the peripheral old phloem died or began collapsing in *C*Las-infected leaves of all the cultivars, some young replacement phloem was generated directly adjacent to the undifferentiated vascular cambium zone as shown above in Figure 3D. Compared to the normal phloem, the newly generated replacement phloem was generally composed of sieve elements, companion cells, and phloem parenchyma cells, but neither phloem fibers nor sclereids were observed. The size of replacement phloem was smaller than the peripheral normal phloem (Figures 3D, 4, 5).

**Figure 6:**
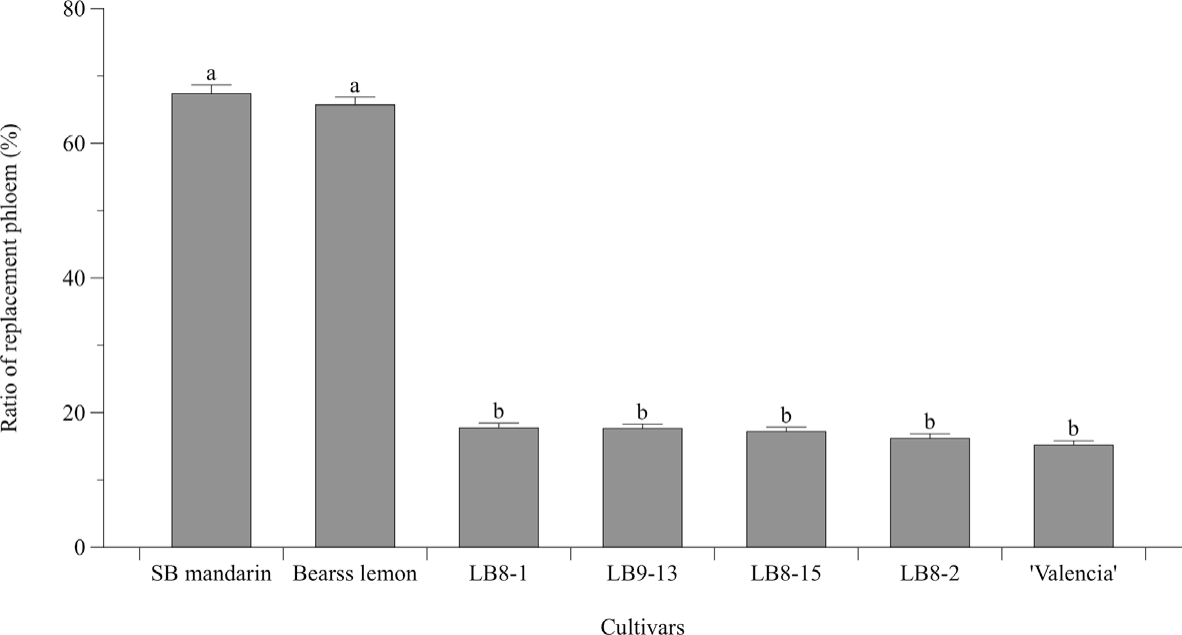
Bar chart showing the ratio (%) of replacement phloem (the replacement phloem width/the total phloem width × 100). Bar and error bar denote means and 95% confidence intervals, respectively. Means that are significantly different in one way ANOVA analysis and Tukey’s HSD test are represented by different small letters above bars (*p* < 0.05).

Statistically speaking, SB mandarin (Figure 4B) and “Bearss” lemon (Figure 4A) had the largest replacement phloem ratio of 67.37 and 65.73%, respectively, which was significantly higher (*p* < 0.05) than in HLB-sensitive cultivars (Figure 6). Viewed in transverse section among all the examined samples, “Bearss” lemon (Figure 4A) and SB mandarin (Figure 4B) had the largest replacement phloem band and cell layer indicating greater activity of the vascular cambium. The replacement phloem in “Bearss” lemon (Figure 4A) and SB mandarin (Figure 4B) likely enables a prolonged actively functioning phloem tissue, with very little necrosis and collapse. Although SB siblings and “Valencia” also had the replacement phloem layer (Figures 4, 6), their newly generated replacement phloem cells become necrotic and showed typical anatomical alterations of HLB disease as described above (Figures 4C–G). Although SB siblings had the largest width of total phloem layer, their newly generated replacement phloem cells become necrotic and showed typical anatomical alterations of HLB disease as described above (Figures 4D–G). Valencia sweet orange had a narrowest ring of newly generated replacement phloem (15.19%) (Figure 6), and the degeneration of replacement phloem is most pronounced (Figure 4C).

Similar to lamina, very little or no starch accumulation was observed in the HLB-free SB mandarin midrib (Figure 3A), while starch accumulation in midribs of sensitive “Valencia” sweet orange (Figures 4C, 5A,B, and Supplementary Figure S1E) and SB mandarin siblings (Figures 4H,J,L,N, and Supplementary Figures S1F–I) was far greater than that in “Bearss” lemon (Figure 4A and Supplementary Figure S1D) and SB mandarin (Figure 4B and Supplementary Figures S1B,C). Spatially, these excessive starch grains in midribs are located analogously to the lamina in the epidermis and mesophyll cells (palisade and spongy parenchyma cells) (Figure 4). Over-accumulation of starch grains in the HLB-sensitive cultivars was also conspicuously found in phloem sieve elements and parenchyma cells, phloem ray parenchyma cells, xylem ray parenchyma cells (Figure 5 and Supplementary Figure S1), pith parenchyma cells (Supplementary Figure S2), and even in tracheids (Figure 4C and Supplementary Figure S2). The pith which is encircled by a ring of xylem consisted of parenchyma cells (Supplementary Figure S2A). Pith parenchyma cells of “Valencia” sweet orange and SB siblings contained an abundance of starch grains (Supplementary Figures S1E–I). The starch grains were also found in “Bearss” lemon (Supplementary Figure S1D) and SB mandarin, but in substantially lower quantities (Supplementary Figures S1B,C).

## Discussion

Anatomical characteristics concerning interior structures are an integral part of the resistance of plant responses to abiotic and biotic stress, contributing to optimization of cultivation practices and selection of new and/or highly productive varieties in stress conditions (Quarrie et al., 2015). Different citrus species varied in responses to *C*Las infection (Fan et al., 2012; Fan et al., 2013). Therefore, the pathological and anatomical responses to *C*Las infection in different citrus varieties could help to identity one of the possible mechanisms underlying the HLB tolerance or sensitivity.

### Less Interior Structural Destruction and Disorganization Caused by *C*Las Infection in “Bearss” Lemon and “LB9-9” Sugar Belle^®^ Mandarin Trees

Here, all the foliage samples from throughout the spring flushes are assumed to have been almost simultaneously subjected to severe *C*Las attack under natural Florida field conditions, within the same geographical location and under common horticultural management. Different cultivars shared some common microscopical manifestations and anatomical aberrations, but they exhibited diverse HLB-associated histopathological symptom severity.

The most notable and common disease manifestation in all the examined cultivars was abundant callose deposition. Our observations showed the increased callose deposition was mainly distributed in the new and replacement phloem area (Figure 5). The preference of distribution in new and/or replacement phloem area supported Achor’s view that plugging is a key primary response to *C*Las infection (Achor et al., 2010) and had some resemblance to the case of American elm (*Ulmus americana* L.) with the frequent occurrence of callose deposition in replacement phloem and sieve tubes close to vascular cambial zone (Braun and Sinclair, 1976). Callose, a β-1,3 glucan, along with PP2 (phloem protein 2) of filamentous appearance, were the main obstructive media directly involved in phloem plugging, instead of the *C*Las bacterial itself, which is not found in sufficient numbers to cause phloem sieve element plugging (Kim et al., 2009; Achor et al., 2010; Fan et al., 2012; Fan et al., 2013; Johnson et al., 2014; Etxeberria and Narciso, 2015). The direct evidence is that the diameter of sieve plate pores of dicotyledons likely ranges from a fraction of 1 to 14 μm (Esau and Cheadle, 1959), while the diameter of *C*Las is from 0.1 to 0.2 μm (Bové, 2006; Wang et al., 2017). The massive callose deposition observed in all the HLB-affected cultivars here corroborated the earlier study that callose deposition plays a role as a defensive fortification of the citrus tree (Kim et al., 2009; Achor et al., 2010) to narrow the connecting strands or seal the sieve pores completely when the flow through sieve tubes becomes detrimental (Esau and Cheadle, 1959).

Another histopathological disturbance caused by *C*Las infection is characterized as phloem necrosis. Although the presence of *C*Las in the phloem was not confirmed, localized phloem necrosis was observed to scatter through vascular tissue of sweet orange leaves (Schneider, 1967; Schneider, 1968). Schneider also stated that occasionally, excessive hypertrophy of ray and phloem parenchyma cells, as well as increased differentiation of vascular cambium, and aggravated phloem tissue necrosis (Schneider, 1967; Schneider, 1968). Reportedly, the phloem necrosis could be associated with an abnormal swelling of the middle lamella between cell walls surrounding sieve elements (Folimonova and Achor, 2010) and misshapen phloem parenchyma cells (Folimonova and Achor, 2010; Brodersen et al., 2014). Here, the amount of phloem necrosis and parenchyma cell hypertrophy in “Valencia” sweet orange and SB siblings were significantly greater than that in “Bearss” lemon and SB mandarin. Further, the necrosis was widely distributed in phloem of “Valencia” sweet orange and the SB siblings, while minimal and infrequent phloem necrosis was found in “Bearss” lemon and SB (Figure 4).

In addition, although phloem collapse with cell wall distortion and thickening as well as cambium degeneration was observed in HLB-affected “Bearss” lemon and SB mandarin leaves, it was much less severe than in “Valencia” sweet orange or the sensitive SB siblings. Partially or entirely collapsed sieve elements and companion cells, even in the newly generated replacement phloem and occasionally disorganized vascular cambium cells, could be easily found in the HLB-sensitive types (Figure 4). The significantly collapsed sieve element and companion cells showed disordered cellular organization with dissolved cytoplasmic structure, as well as both cytoplasmic and cell wall thickening, while phloem parenchyma cells were expanded to turgid size (Figure 5). According to Schneider’s earlier study, phloem collapse was primarily due to hyperactive differentiation of vascular cambium and hypertrophy of parenchyma cells surrounding the necrotic phloem pocket (Schneider, 1968). Folimonova and Achor (2010) demonstrated that swelling of middle lamella between cell walls surrounding sieve elements potentially has been attributed to phloem collapse and necrosis.

The interior structural destruction and disorganization may contribute to an inhibition of source-to-sink transportation of photosynthetic products, as well as carbohydrate metabolism imbalances (Koh et al., 2012; Etxeberria and Narciso, 2015). As a consequence, the carbohydrate metabolism imbalance was manifested as over-accumulation of starch in the HLB-affected lamina (Figure 2) and midribs (Figures 4, 5 and Supplementary Figures S1,S2). The unusual excessive starch distribution presented here corroborated the previous pathological observations that not only the photosynthetic mesophyll cells, vascular parenchyma cells and sieve elements but also epidermal and pith parenchyma cells were replete with starch granules (Schneider, 1968; Etxeberria et al., 2009; Achor et al., 2010; Folimonova and Achor, 2010; Gonzalez et al., 2012). In addition to the reported intracellular distribution, our anatomical results described the occurrence and distribution of starch accumulation in xylem tracheid elements (Supplementary Figures S1H,S2). In this present work, starch accumulation was also detected in xylem, which may be indicative of the xylem tissue as a starch deposition site and the newly formed xylem elements as strong sinks (Drossopoulos and Niavis, 1988).

Excessive starch granule content disrupts the internal thylakoid structure of the chloroplasts, which likely explains why HLB-affected leaves have an externally visible symptom of an asymmetrical chlorosis referred to as “blotchy mottle appearance” (Bové, 2006; Achor et al., 2010; Fan et al., 2013). Here, the excessive starch accumulation was also in conformity with evidence of up-regulation of starch biosynthesis encoding genes such as ADP-glucose pyrophosphorylase, starch synthase, granule-bound starch synthase, and starch debranching enzyme (Kim et al., 2009; Aritua et al., 2013), and down-regulation of functional genes related to starch breakdown process such as *DPE*2 and *MEX*1 (Fan et al., 2010). In HLB-affected samples from “Bearss” lemon and SB mandarin, starch accumulation was much less abundant than in the sensitive types.

The results presented in this work indicate that, although physically, morphologically and pathologically similar, “Bearss” lemon and SB mandarin were much less affected anatomically and structurally by *C*Las than “Valencia” sweet orange and the SB siblings; the latter also suffered more severe phloem destruction and disorganization. Internal structural preservation of the tolerant cultivars generally was superior to the sensitive types when under *C*Las pathogen attacks.

### More Replacement Phloem Generation to Compensate for the Dysfunctional Phloem in “Bearss” Lemon and “LB8-9” Sugar Belle^®^ Mandarin Trees

In the HLB-affected citrus samples, the vascular cambium became hyperactive to generate a wide band of replacement phloem (Figures 3D, 4, 5). The replacement phloem consisted of the assemblages of sieve elements, companion cells, and phloem parenchyma cells, but lacked phloem fibers, and the size of the replacement band was less than the peripheral old phloem. In the HLB-affected samples of “Bearss” lemon and SB mandarin, most of the phloem cells that were not totally damaged or collapsed by *C*Las infection were likely still functional. Before any evidence of massive phloem necrosis and its eventual collapse, there has been considerable replacement phloem regenerated from the cambial differentiation zone in these tolerant cultivars (Figure 4). However, for the HLB-affected samples of “Valencia” sweet orange and SB siblings, phloem degeneration could occur in the peripheral old phloem of the tissue and proceeded toward the newly generated replacement phloem, even the undifferentiated vascular cambial zone. The newly generated replacement phloem rapidly became necrotic, which led to collapse (Figures 4, 5).

Such an analogous situation has also been reported in elm phloem necrosis with the production of new and replacement vascular phloem tissue (Braun and Sinclair, 1976). In citrus, Schneider (1968) found some necrotic but some still functional sieve tubes within the replacement phloem in HLB-affected sweet orange, corresponding to the anatomaical observations here of newly generated replacement phloem, but necrotic and/or collapsed in HLB-sensitive types (Figures 4, 5). According to Albrigo et al. (2014), HLB-affected samples from both young potted and field-grown trees generated more layers of new phloem cells when compared to samples from healthy control trees. The newly produced ring of metaphloem from HLB-affected citrus seems to be healthy phloem functionally in both petioles and stems (Brodersen et al., 2014). Fan et al. (2012) also reported significantly up-regulated genes involved in cell wall biosynthesis in HLB-affected rough lemon, potentially supporting the formation of new functional phloem tissues, which potentially explains one of the tolerance mechanisms of rough lemon to HLB.

“Bearss” lemon and SB mandarin produced significantly more replacement phloem (Figure 6), and this newly generated replacement phloem was also less affected than in the sensitive types. These traits can be regarded as adaptive and compensative to the adverse effects of *C*Las infection, contribute to the mitigation of phloem dysfunction, and help to support and maintain phloem transport longer.

### Pathological Anatomical Mechanisms Underlying HLB Tolerance of “Bearss” Lemon and “LB8-9” Sugar Belle^®^ Mandarin Trees

HLB-affected trees have a compromised and dysfunctional phloem system. To overcome the compromised phloem, significantly more replacement phloem in HLB-tolerant cultivars was generated. The lower starch accumulation in SB mandarin and “Bearss” lemon may be a consequence of greater development of replacement phloem. As a consequence of regenerating functional phloem, the transport of photosynthates is less obstructed, and less starch accumulation is observed in SB mandarin and “Bearss” lemon. The lower levels of phloem disruption and greater phloem regeneration are two key elements that contribute to HLB tolerance in these diverse citrus cultivars. Further support of this conclusion comes from a report by Zheng et al. (2018) and Etxeberria (unpublished data, from the Final Report of CRDF Proposal #899). They demonstrated that Strigolactone (SL) applications to HLB-affected “Hamlin” sweet orange trees successfully ameliorated the adverse effects of HLB. They observed an SL-induced vascular system re-establishment, and claimed this to be one reason for the reversal of HLB symptoms, including the disappearance of starch accumulation.

Earlier *C*Las graft inoculation experiments conducted under controlled greenhouse successfully showed the high HLB tolerance of the commercial lemon cultivars, based on the minimal symptoms and continuous growth (Folimonova et al., 2009), which may be extrapolated to field performance. Similarly, in Florida citrus plantings, HLB-affected lemon or lime-like phenotypes performed better than many other citrus, with little leaf loss and the densest canopies to sustain greater growth (Ramadugu et al., 2016; Miles et al., 2017).

According to the experience of Florida citrus growers, SB mandarin and “Bearss” lemon trees, even though displaying obvious blotchy mottle symptoms on older leaves later in the season, still maintained productivity and kept quite vigorous with full canopies, when the trees were maintained with good canopy and crop load management, and proper water and nutrient management (Gmitter, 2017). Previous continued and substantial field evaluations have also shown that SB mandarin trees can endure and thrive despite *C*Las infection and typical HLB symptoms (Stover et al., 2016). Recent reports of volatile and nonvolatile metabolomics associated the possible HLB tolerance of SB mandarin with several compounds contributing to anti-microbial activity, to withstand pathogen attack (Killiny et al., 2017). From the gene expression level, there are a large number of differentially expressed genes comparing SB with the more sensitive “Clementine” mandarin, among which the most enriched GO term is secondary cell wall biogenesis (Deng et al., unpublished data), which also well supports the conclusions of this anatomical study.

## Conclusion

This study presents an abundance of evidence of decreased phloem destruction, together with more replacement phloem generation, that underlies the greater HLB-tolerance of “Bearss” lemon and LB8-9 Sugar Belle^®^ mandarin, in field grown trees naturally exposed to HLB. These cultivars represent genetically diverse citrus species, but the anatomical responses associated with tolerance are the same. Together with previous reports, these new detailed observations provide more evidence of an anatomical basis for some aspects of HLB tolerance, which will hopefully ultimately lead to some new approaches to combat HLB in the near future.

## Author Contributions

FG conceived and designed the study and revised the manuscript. HD performed the experiment and wrote the manuscript. DA, EE, DS, and QY helped to revise the manuscript. DA also contributed the staining methods. DS provided technical guidance using microscope and protocol. EE also provided helpful discussions, aided with interpretation of results and helped to arrange the structure of the manuscript to make it a more solid conclusion. GL, DD, and QY helped to conduct data analysis. All authors approved the final manuscript.

### Conflict of Interest Statement

The authors declare that the research was conducted in the absence of any commercial or financial relationships that could be construed as a potential conflict of interest.

## Supplementary Material

SUPPLEMENTARY FIGURE S1 Starch grains in pith parenchyma cells of midrib tisssue. (**A**) HLB-free SB mandarin, showing no starch accumulation; (**B**,**C**) SB mandarin; (**D**) ‘Bearss’ lemon; (**E**) ‘Valencia’ sweet orange; (**F**) LB8-1 mandarin; (**G**) LB8-2 mandarin; (**H**) LB8-15 mandarin (Starch grains in the vessels are visible); **(I)** LB9-13 mandarin. The pores in pith parenchyma cells are plasma membrane-lined pores that are responsible for spanning the adjoining cell walls to permit intercellular passage of molecules and signals.

SUPPLEMENTARY FIGURE S2 Epifluorescence photomicrographs of transverse sections of LB8-2 mandarin showing the high accumulation of starch spatially located in phloem sieve elements and parenchyma cells (**A,B**), xylem ray parenchyma cells (**B**, the red arrow indicated), tracheids elements and vessel elements (**B**, the red rectangle frame indicated), as well as the pith parenchyma cells. Scale bars = 50 µm.
