# Supplementary figures and images for: Phloem Regeneration Is a Mechanism for Huanglongbing-Tolerance of “Bearss” Lemon and “LB8-9” Sugar Belle® Mandarin
Source: Front Plant Sci. 2019 Mar 20;10:277. doi: 10.3389/fpls.2019.00277 (PMC6435995; doi:10.3389/fpls.2019.00277)

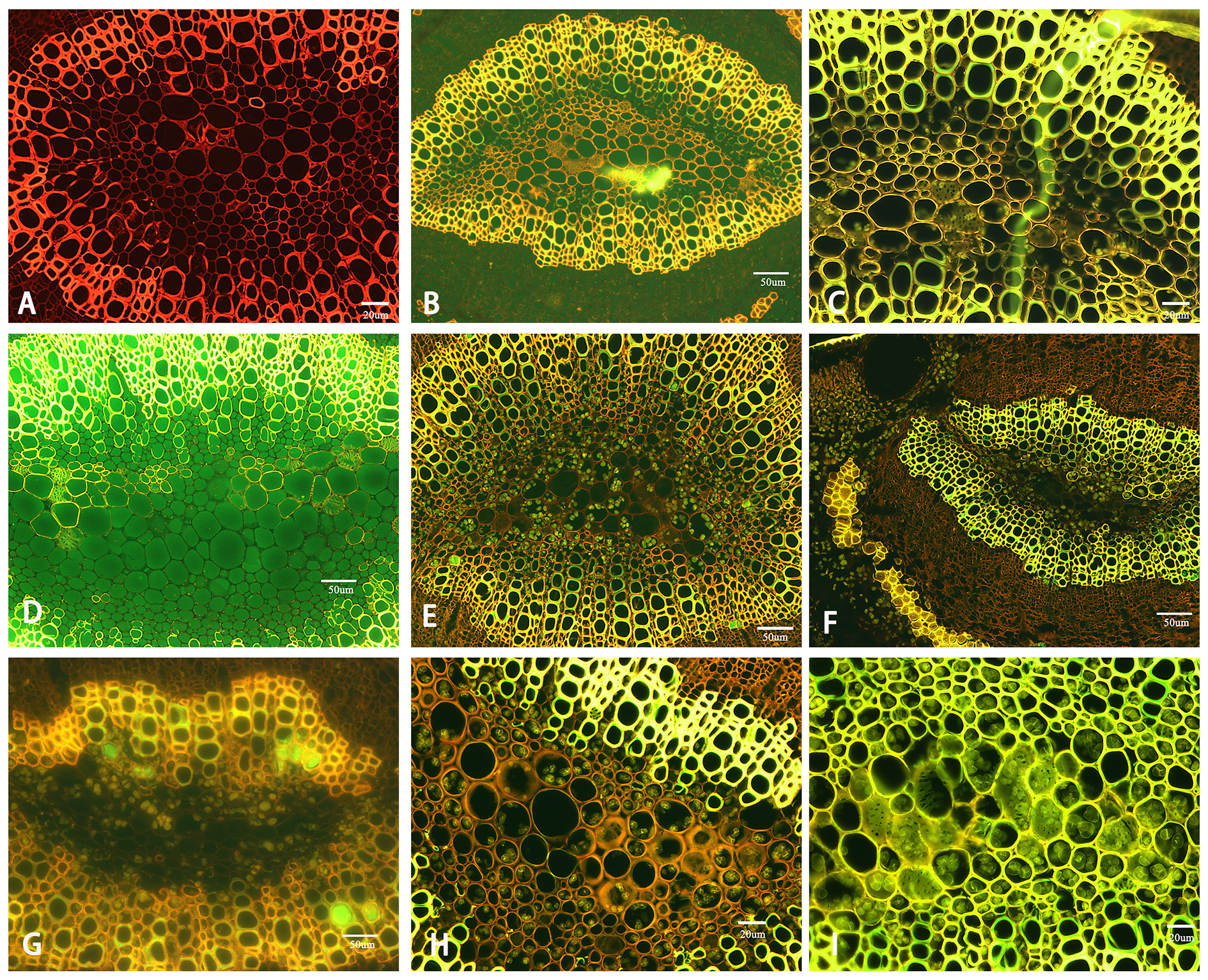

Supplement: SUPPLEMENTARY FIGURE S1 — Starch grains in pith parenchyma cells of midrib tisssue. (A) HLB-free SB mandarin, showing no starch accumulation; (B,C) SB mandarin; (D) ‘Bearss’ lemon; (E) ‘Valencia’ sweet orange; (F) LB8-1 mandarin; (G) LB8-2 mandarin; (H) LB8-15 mandarin (Starch grains in the vessels are visible); (I) LB9-13 mandarin. The pores in pith parenchyma cells are plasma membrane-lined pores that are responsible for spanning the adjoining cell walls to permit intercellular passage of molecules and signals. [file Image_1.TIF]

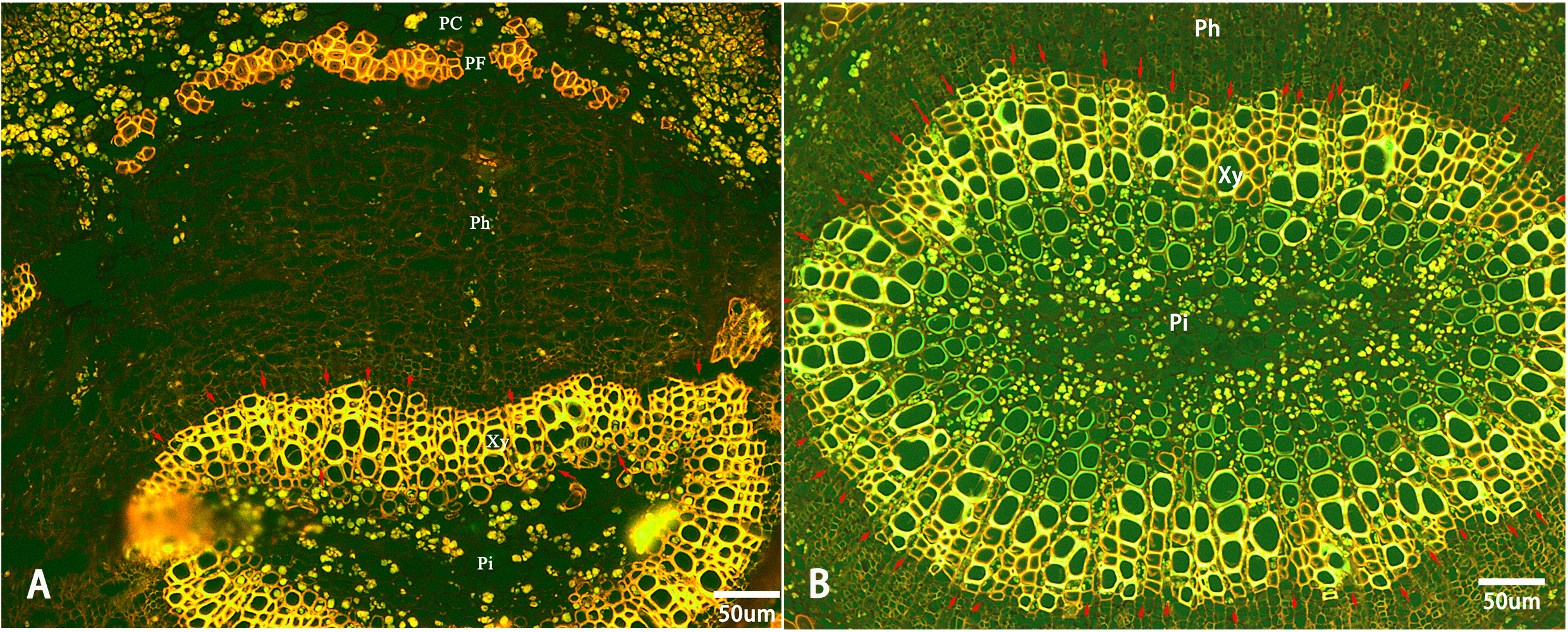

Supplement: SUPPLEMENTARY FIGURE S2 — Epifluorescence photomicrographs of transverse sections of LB8-2 mandarin showing the high accumulation of starch spatially located in phloem sieve elements and parenchyma cells (A,B), xylem ray parenchyma cells (B, the red arrow indicated), tracheids elements and vessel elements (B, the red rectangle frame indicated), as well as the pith parenchyma cells. Scale bars = 50 µm. [file Image_2.TIF]
